# Supplementary material for: The Use of Electrochemical Impedance Spectroscopy as a Tool for the In-Situ Monitoring and Characterization of Carbon Nanotube Aqueous Dispersions
Source: Nanomaterials (Basel). 2022 Dec 12;12(24):4427. doi: 10.3390/nano12244427 (PMC9786001; doi:10.3390/nano12244427)
Supplement: Supplementary file 1 [file nanomaterials-12-04427-s001.zip › nanomaterials-2042404-supplementary.pdf]

nanomaterials

Supplementary Material

Article

The Use of Electrochemical Impedance Spectroscopy as a Tool for the In-Situ Monitoring and Characterization of Carbon Nanotube Aqueous Dispersions

Aikaterini Gkaravela, Ioanna Vareli, Dimitrios G. Bekas, Nektaria-Marianthi Barkoula, Alkiviadis S. Paipetis *

Department of Materials Science and Engineering, University of Ioannina, 45110, Ioannina, Greece
* Correspondence: paipetis@uoi.gr; Tel.: +3026510-08001

Calculation of Conductivity

The conductivity is the inverse of the Impedance measure. The real and imaginary part $Z'(\omega)$ & $Z''(\omega)$ of the Impedance Z can be employed to calculate the conductivity in the frequency domain via the following equation.

$$\sigma(\omega)=\left(\frac{Z'(\omega)}{Z'(\omega)^2+Z''(\omega)^2}\right)k \quad (S1)$$

where k is the geometrical constant of the material under testing.

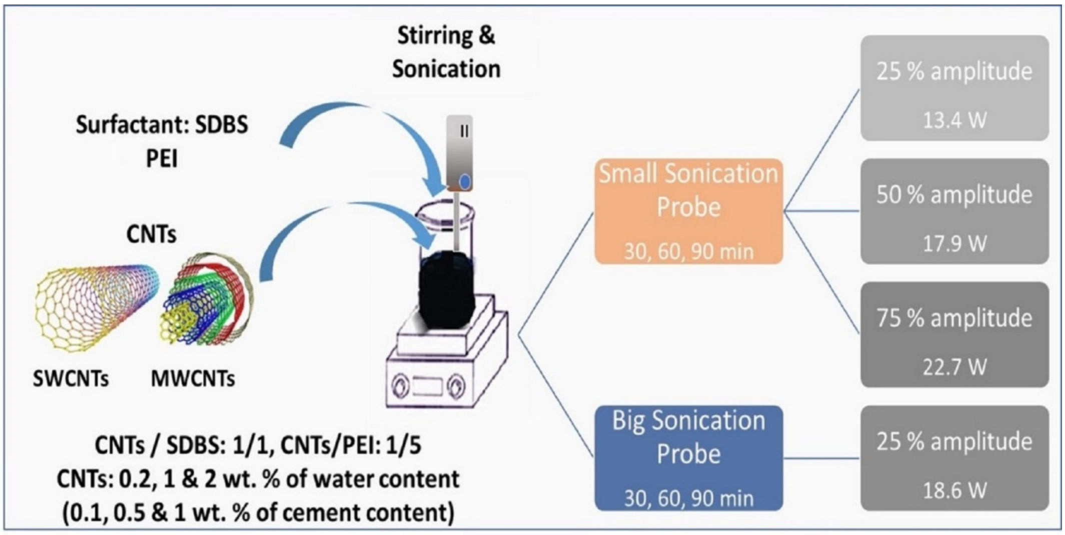

Figure S1. Schematic representation of the dispersion combinations and sonication process.

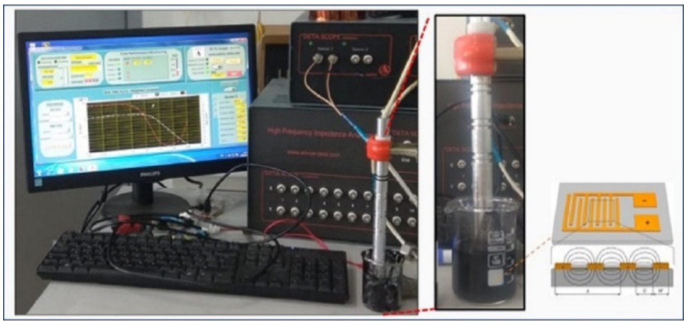

Figure S2. Experimental set-up of the EIS measurement during sonication.

In Fig. S3, the EIS plots of ǀZǀ of MWCNTs dispersions at low sonication amplitude (i.e., 25 %) are presented. As observed in Fig. S3a, at 0.2 wt.% MWCNTs content, the Impedance ǀZǀ presents very high values, which for the low frequencies of the diagram are considered unreal as they are within the set-up measurement limits. Furthermore, no changes are observed in ǀZǀ as a function of sonication time. This can be explained by the fact that the amount of added MWCNTs is below the percolation threshold [52], and an electrically conductive network is not formed, even if the dispersion of MWCNTs is adequate. When MWCNTs are added at 1 wt. % in the solution (Fig. S3b), the ǀZǀ values after 30 and 60 min of the sonication are like those observed previously. After 90 min of the sonication process, a drop of ǀZǀ by seven orders of magnitude is observed, followed by a linear reduction. This drop indicates the formation of a continuous conductive network. Interestingly, at 2 wt. % MWCNTs, the very high ǀZǀ values suggest the absence of a conductive network, although the content of MWCNTs is above the percolation threshold. Based on the above, it can be deduced that the sonication power provided by the small sonication probe at an amplitude of 25 % is insufficient to disperse 2 wt. % MWCNTs even after 90 min of sonication.

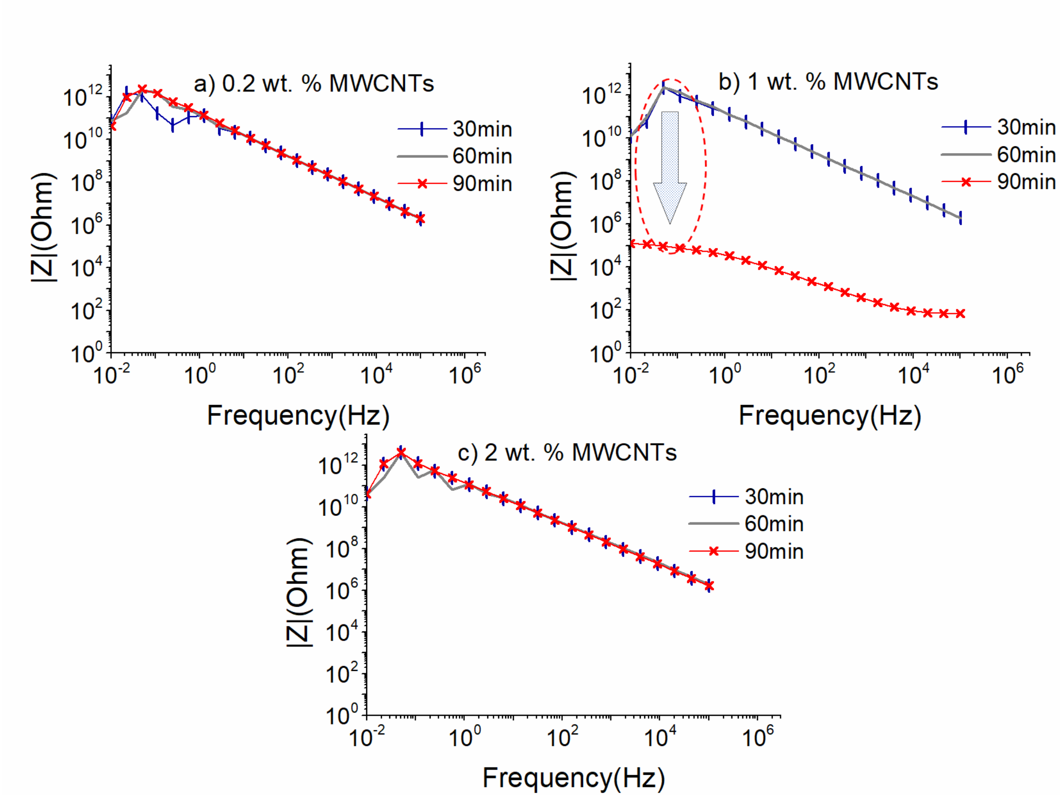

Figure S3. EIS plots of aqueous dispersion with a) 0.2, b) 1, and c) 2 wt. % MWCNTs, using the small sonication probe at an amplitude of 25 % (13.4 W).

The dispersion behavior for MWCNT contents above the percolation threshold (2 wt. %) at 50 % amplitude is presented in Fig. S4. At 2 wt.% MWCNTs, the conductive network is formed after 60min of the sonication process. The striking in this case is a sharp increase in the ǀZǀ magnitude that appears after 90min of the sonication process. This behavior indicates a degradation of the CNTs and/or SDBS structure and destruction of the formed electrical network. It is speculated that, due to the high concentration of CNTs/SDBS, 90 min of ultrasonication results in a separation of the two phases, leading to re-agglomeration of the CNTs, as illustrated in Fig. S5. Concurrently, excessive sonication could result

in the degradation of the aspect ratio of the MWCNTS and in turn their capability to form a conductive network even at 2 wt. % content.

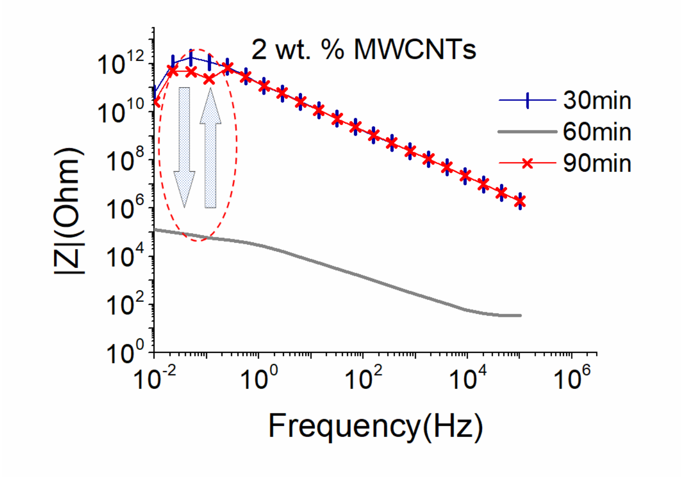

Figure S4. EIS plots of aqueous dispersion with 2 wt. % MWCNTs, using the small sonication probe at an amplitude of 50 % (17.9 W).

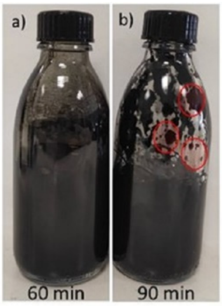

Figure S5. Dispersion of 2 wt. % MWCNTs after a) 60 min and b) 90 min of sonication, using the small sonication probe at 50 % amplitude (17.9 W).

This phenomenon is further demonstrated in the case of solutions with 1 wt. % MWCNTs (see Fig. S6), where an additional increase of the ultrasonic power at 22.7W (75% amplitude) does not result in the formation of a conductive network at any dispersion duration. It is believed that the high power applied to the solution destroys the structure of the nanotubes from the early stages of the ultrasonic process resulting in deterioration of the aspect ratio of the CNTs and, in turn, in very high impedance |Z| values.

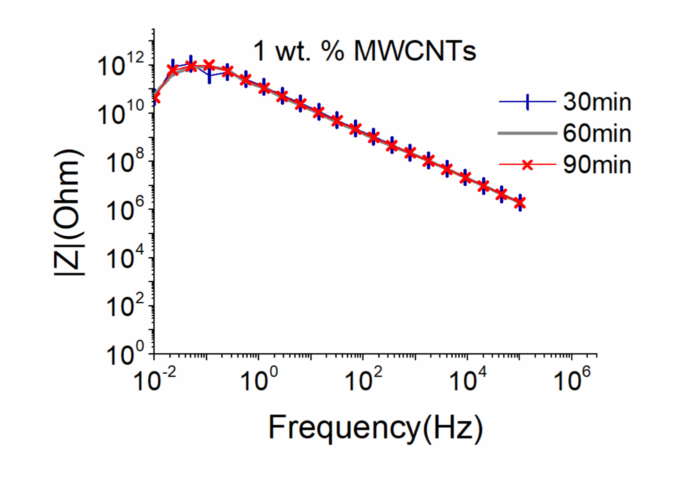

Figure S6. EIS plots of aqueous dispersion with 1 wt. % MWCNTs, using the small sonication probe at an amplitude of 75 % (22.7 W).

For further support in the study of the effect of the sonication power on the dispersion ability of the SWCNTs, EIS measurements were conducted in aqueous dispersion with 1 wt. % SWCNTs, using the small sonication probe at the amplitude of 25% (Fig.S7).

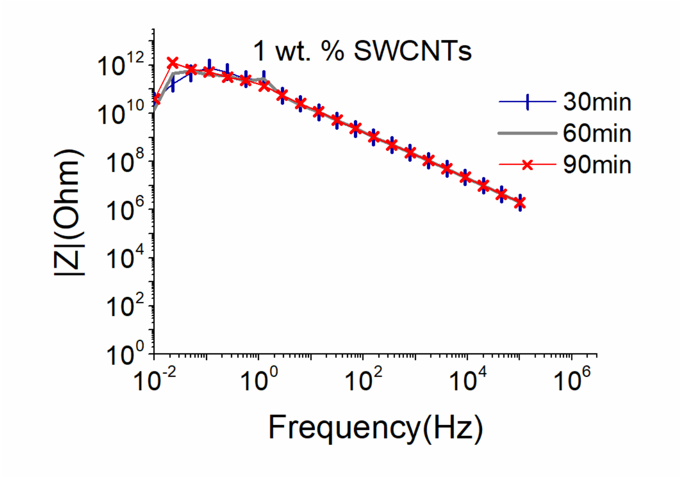

Figure S7. EIS plots of aqueous dispersion with 1 wt. % SWCNTs, using the small sonication probe at an amplitude of 25% (13.4 W).

As presented and discussed in the main manuscript (Fig. 1b), the small sonication probe is insufficient to disperse the SWCNTs in an amplitude of 50%; therefore, it is obvious that even lower sonication power results in similar behavior.

The dispersion efficiency of solutions with 0.2 and 2 wt. % MWCNTs is assessed in Fig. S8.

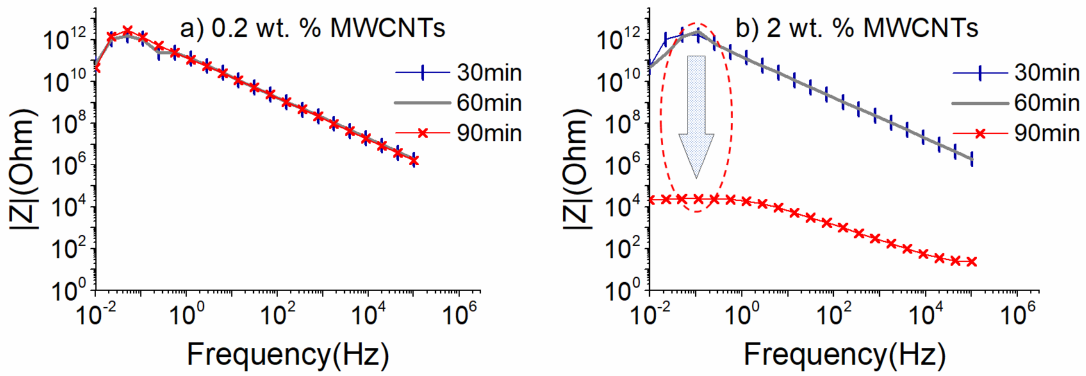

Figure S8. EIS plots of aqueous dispersion with a) 0.2 and b) 2 wt. % MWCNTs, using the big sonication probe at an amplitude of 25 % (18.6W).

As expected from the results of the small probe, the values of ǀZǀ remain high in Fig. S8a, even after a total sonication duration of 90 min, since the level of CNT reinforcement is below the percolation threshold. On the other hand, for the dispersions with 2 wt. % MWCNTs, a drop of the impedance ǀZǀ by approximately eight orders of magnitude is obtained after 90min of ultrasonication, indicating that a conductive path is formed only after excessive sonication (Fig. S8b).

Fig. S9 presents the dispersion ability of the SWCNTs using the big sonication probe. At 0.2 wt. % of SWCNTs and 60 min of sonication, a conductive network is formed, represented by a drop of ǀZǀ by seven orders of magnitude (Fig. S9a). Although this content is considered below the percolation threshold in the case of MWCNTs, here it is verified that SWCNTs differ in their electrical conductivity properties and can form, at lower contents, a continuous network if successfully dispersed. EIS response is the same as in the case of 1 wt. % that is presented in the main manuscript. Interestingly, for dispersions with 2 % SWCNTs, the network is formed within the first 30 min of sonication, and additional sonication leads to even lower ǀZǀ values (Fig. S9b).

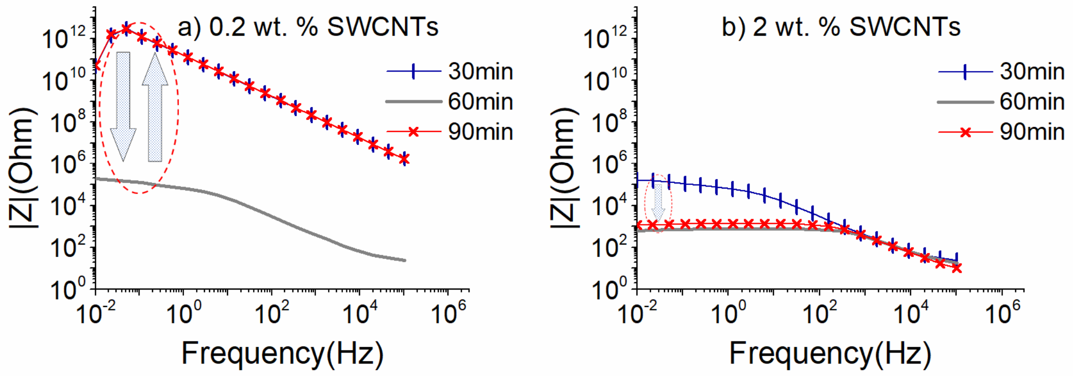

Figure S9. EIS plots of aqueous dispersion with a) 0.2 and b) 2 wt. % SWCNTs, using the big sonication probe at an amplitude of 25 % (18.6W).

The dispersion ability of the MWCNTs during the presence of PEI with 2 wt. % is presented in Fig S10. Effective dispersion is achieved after 60 min of sonication (30 min earlier compared to solutions without PEI – see Fig. S8b), and thus, it can be concluded that the presence of PEI overall facilitates the dispersion process of MWCNTs.

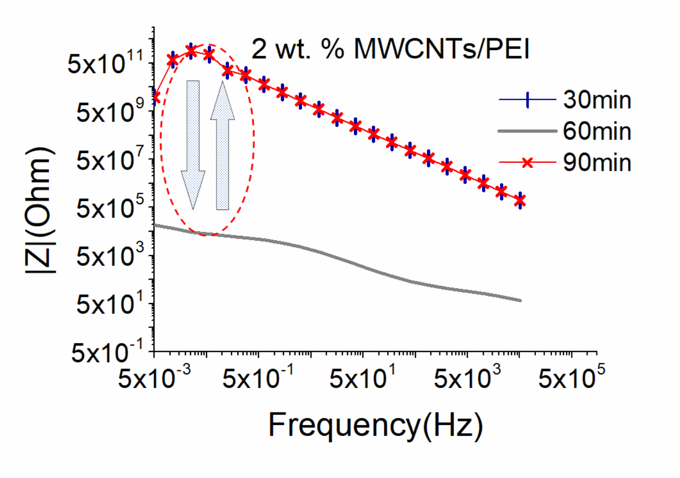

Figure S10. EIS plots of aqueous dispersion with 2% MWCNTs, using the big sonication probe at an amplitude of 25 %, in the presence of PEI (18.6 W).

To support the comparison of the dispersion ability of the different experimental combinations presented in the main manuscript in Fig. 8, the results of the additional concentrations of CNTs are presented in Fig S11. As observed in Fig. S11a, a conductive network is not formed at 0.2 wt.% MWCNTs, for a range of sonication energies between app. 20 – 100 kJ. On the contrary, intermedium sonication energies, between 60 and 80 kJ, are required for the effective dispersion of 0.2 wt. % SWCNTs (Fig. S11b). Despite their obvious similarities, SWCNTs, and MWCNTs differ significantly in their physical properties due to their structural differences. Thus, as confirmed by the EIS measurements, it is possible to create a conductive network with as low as 0.2 wt.% SWCNTs. Additionally, medium to high sonication duration is required for the dispersion of 2 wt. % MWCNTs, as observed in Fig. S11c. Interestingly, at 2 wt. % SWCNTs (Fig. S11d), a conductive network is formed even after applying low levels of sonication energy (33.5 kJ). This behavior suggests that even if SWCNTs are not fully dispersed in this case, their amount is quite high, resulting in a conductive network of better and less dispersed SWCNTs.

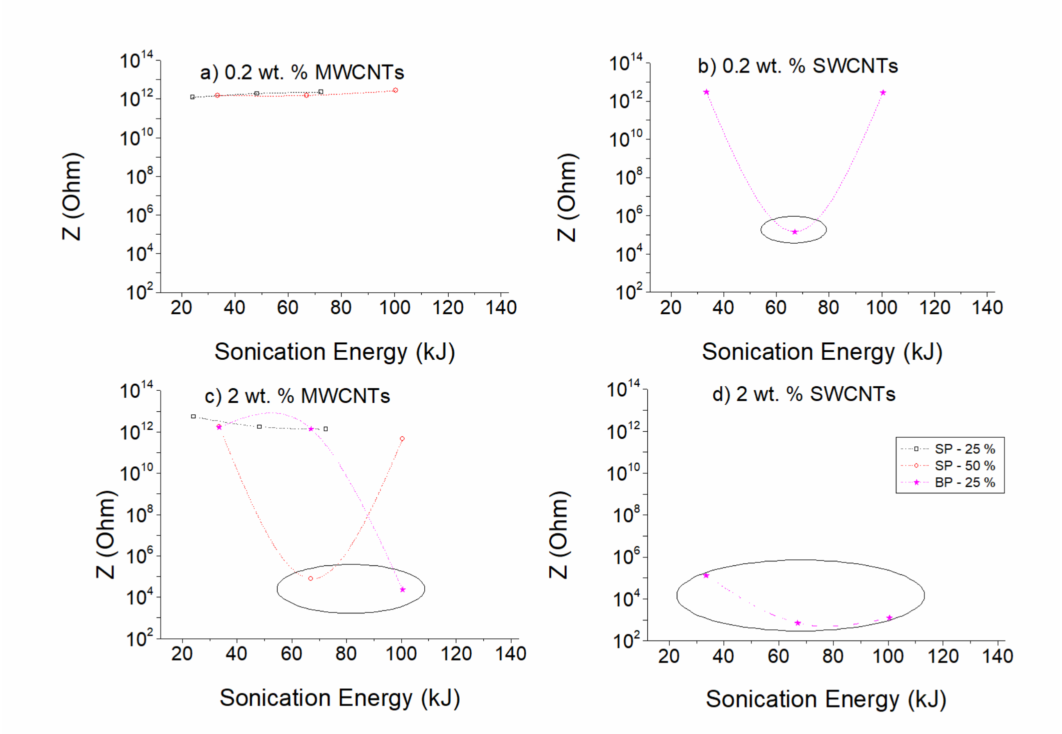

Figure S11. Variation of ∣Z∣ values at a frequency of 0.05Hz as a function of sonication energy of aqueous dispersions of MWCNTs a) 0.2 wt. %, b) SWCNTs 0.2 wt. %, c) MWCNTs 2 wt. %, and d) SWCNTs 2 wt. %. Note that SP stands for small and BP for big probe; P stands for PEI.
